# Surgical Cliniques

**Published:** 1868-04

**Authors:** J. C. Hughes


Medical Department Iowa State University.

BY PROF. J. C. HUGHES, M. D.

Among the many interesting cases which have presented themselves for operation and treatment before the Medical Class of the session of 1867–68, we shall only mention a few, which to our mind will most interest the readers of the JOURNAL:

Mr. C———, aged 36, in May last, was attacked with hernia, (*oblique inguinal*), which became strangulated. The physician called, satisfied with the diagnosis, immediately applied the taxis, and reduced, as he supposed, the protruding gut. The symptoms however returned, and within twenty-four hours he was again summoned to relieve the sufferings of his patient. The taxis was again applied and the tumor reduced, but without the relief which had attended the first operation. The patient continued to suffer at times for several days, when increased symptoms of strangulation again presented themselves. I was called, in company with Prof. Carpenter, to see the patient, and after a careful examination we were satisfied that adhesions existed, and that the case was one of irreducible hernia. We administered paliatives with hope of relief, but the symptoms of strangulation did not yield, and next morning I proceeded to operate. Having made a longitudinal incision over the tumor, I made a careful dissection of the integument and facia, found the sack adherent and the intestine to the extent of one and a half inches gangrenous, and at two points sphacelus had actually occurred. Nothing better now presented for the patient than the formation of an artificial anus. This having been accomplished, the point of stricture relieved, the patient was comparatively comfortable. The fæcal discharges taking place from the new opening, without the controlling influence of a proper sphincter, was, to say the least, very annoying to the patient. To obviate some of the difficulties which from time to time presented, the patient thinking that the deficiency of one and a half inches of the intestine might be made up, conceived the idea of substituting a silver tube five-eighths of an inch in diameter, and of sufficient length to have each extremity retained within the open points of the intestine. This he had constructed and applied according to his own idea. The instrument not fitting completely, allowed the discharge to escape, and the want of peristaltic action in the tube prevented it from taking the place of the original. The fæcal matter from above forced the entire tube into the lower opening and the instrument disappeared beyond reach into the intestinal canal. Imagine the anxiety of the patient for four long days, when to his great surprise and gratification the instrument made its appearance at the anus. Not satisfied with the experiment, he increased the size of each extremity, and affixed a pin one inch or more in length to its surface, preventing even the possibility of its escape into the lower bowels. The tube again introduced did not answer the ends for which it was intended, and I was consulted in relation to an operation for the cure of the difficulty. Here was an artificial anus connected with the scrotum and involving the large intestine. The chance for withdrawal of the protruded parts within their natural cavity could not be hoped for. There was not presented the two openings in close apposition with its intervening septum, as in ordinary cases of strangulation where the knuckle of intestine presents. Had this been the case, we would have had no trouble as to the course to be pursued. But the case presented thus: The opening one and a half inches long, occupying the side and half of the intestine's girt, with adhesions preventing approximation of its points of opening. A question which naturally presented itself was, will half the girt, if brought together, constitute a sufficient tube for the healthy performance of the various functions belonging to the canal; or would the stricture thus induced lead to more serious results than at present exists? I decided to operate, and having presented the patient before the class, pared the edges of the gut, extending the cut surface into the more dense structures until the opening was completely surrounded. The surfaces were now brought together by the hare lip suture and treated for adhesion. The contraction of the gut, the accumulation of gases in the upper canal forced a yielding of the soft structures, followed by sloughing, and the operation failed. A second operation was performed with the same results. Feeling that so much constriction of the natural canal could not be maintained, I determined to enlarge the surface incised, paring integuments outside of the intestines, and then bringing the pared surfaces together, convert a portion of integument into a mucous canal. This was done, and the success attending it, although not complete, has been very satisfactory. The opening is lessened over one-half, the discharges are under the control of the patient. By placing his finger over the opening he can have the discharge take place *per via naturalis.*

Case Second.—Mr. C———, aged 37, occupation a carpenter, on the night of Nov. 19th was struck by some unknown person, inflicting a severe wound over the *supra orbital* ridge of the left side, also an extensive bruise over the right ear, producing symptoms of severe concussion. The patient was brought to my office by the Marshall and one of the city police about 9, P. M. I examined his head, not discovering any fracture, dressed the wound, and ordered a cathartic, requesting a report of his condition in the morning. I heard nothing from the case for nearly three weeks, when his wife called upon me, stating that her husband whose head I had dressed had been suffering for several days from severe pain about the right ear, and had become so stupid that he could scarcely be roused, and wished me to see him. I learned that Dr. Davis, the physician for the city poor, had visited him several times, but was not present on my arrival. I examined the patient carefully, and satisfied myself that he was suffering from compressed brain; but as to the point of compression I was not fully decided. He had suffered from febrile symptoms for several days, complained of great pain in the right temporal region, locating it at the point where the blow had been received. No discharge had taken place from the ear, nor was there any thing to mark the location of the agent of compression. I prescribed alterative doses of mercury with hot applications to the extremities, thinking it possible that a favorable change might take place. Twenty-four hours after found increased coma; stertorous breathing, with other diagnostic symptoms of compression. What was now to be done? The patient rapidly sinking, without any hope of the brain accommodating itself to the compressing agent. Was the compression at the point or points struck, or at the opposite that of *contrecoup*? The only hope left was from the skillful use of the trephine at the point of compression, and the certainty of finding that point extremely doubtful. I gave the family my opinion, urging the necessity of the operation, to which they consented. The house was quite small, and I suggested having the patient conveyed to the Medical College for operation, which met with opposition; but when told that he was insensible, and would not experience any pain from the operation, and that I would have him brought home as soon as operated upon, the family consented. I at once had him placed upon a litter, and in a few minutes he was upon the operating table, where I had an opportunity of exhibiting to the class one of the most interesting cases connected with injuries of the cranium.

Where shall I apply the crown of the trephine? was the next question to be answered. Echo answers where?

I remembered having felt a little roughness over the edge of the frontal bone when examining the wound which I had dressed three weeks before, yet nothing but the scar was left to mark tne spot. Might we not have punctured fracture with spicula of bone, penetrating or otherwise injuring the brain, followed by inflammation and its products? I asked myself. The answer would naturally suggest another inquiry. Why should the patient have suffered from so much pain in the vicinity of the right ear, if the difficulty existed in connection with the left supra-orbital ridge? Yet with this strong evidence before me, I determined to strike the first blow over the frontal bone near the old line of wound. Having made a crucial incision, I dissected up the soft parts exposing the bone, and at a point a little above the supra-orbital ridge, and about one and a half inches from the median line, I placed the crown of the largest sized trephine. The operation was now commenced, and soon one side of the instrument entered the frontal sinus. I now removed the portion of bone forming the outer layer of the sinus, when I at once discovered that injury had been done the frontal bone just at the upper edge of the sinus. I continued the operation until the inner table was removed, which proved the suspicion which had induced me to operate at this point. Here were spicula of bone only two or three lines in thickness, yet sufficient to excite inflammation in the brain, the product of which was beneath the *dura mater*, rendering it necessary to puncture the membranes. This relieved the aggravated symptoms, the patient being at once aroused to consciousness, and before removing him from the operating table he responded to questions asked him. During the operation he was not conscious of any pain. The wound was dressed and he was at once removed to his home, where, assisted by my young friend, Dr. Jenkins, he was closely watched, each day furnishing evidence of marked improvement. No unpleasant symptoms presented, and at this writing he is about his room enjoying the society of family and friends.

Case Third.—L. S., aged 11 years, a daughter of Chas. S., one of our German citizens, has been a great sufferer for the last four years from disease of the femor of the left side. The patient was presented to the Clinique by Dr. Wiseman, the physician of the family, was examined in presence of the class, and gave convincing proof of the existence of necrosis, involving the shaft of the bone for nearly half its length. The soft parts upon the outer surface of the shaft nearly its entire length gave evidence of numerous openings, which had existed from time to time for the discharge of pus or portions of exfoliated bone. But two openings now existed; the outer, two or more inches above the external condyle, the other on the inner surface about the point of connection between the upper and middle third, in close proximity to the femoral artery. Upon introduction of the probe into either opening extensive disease of the bone was elicited, and I decided to operate. The patient was brought under the influence of chloroform, when I made an extensive incision from the opening above the condyle, extending upwards on a line with the shaft for three inches, freely exposing the bone. By this incision I was enabled to learn the extent of the disease, which I found involved the entire shaft for a distance of about four inches. Enlarging the *cloacæ* with a chisel, I introduced the bone forceps and removed several fragments. Finding the opening too small for extraction of the remaining sequestrum, I made an opening upon the inside at the point of discharge referred to, but its proximity to the artery prevented extensive incisions. I was, however, enabled by this incision to remove a portion of the shaft which remained, and so lessen its bulk as to allow extraction from the first opening. The operation being completed, the wounds were filled with lint and the parts approximated by adhesive plaster and bandaged. This case, although showing extensive disease, and requiring a severe operation, is fast convalescing, and I doubt not will make a good recovery.

We might continue our clinical report, and include many interesting cases of disease of the Eye and Ear, which have presented themselves for operation and treatment; cases of Urinary Calculi, both in the male and female; the operation by cutting, and that of lithotripsy; but we must withhold any thing further for the present, and give room for reports from the members of our State Medical Society.

